# Evaluating a transparent coating on a face shield for repelling airborne respiratory droplets

**DOI:** 10.1063/5.0073724

**Published:** 2021-11-11

**Authors:** Bibek Kumar, Sanghamitro Chatterjee, Amit Agrawal, Rajneesh Bhardwaj

**Affiliations:** Department of Mechanical Engineering, Indian Institute of Technology Bombay, Mumbai 400076, India

## Abstract

A face shield is an important personal protective equipment to avoid the airborne transmission of COVID-19. We assess a transparent coating on a face shield that repels airborne respiratory droplets to mitigate the spread of COVID-19. The surface of the available face shield is hydrophilic and exhibits high contact angle hysteresis. The impacting droplets stick on it, resulting in an enhanced risk of fomite transmission of the disease. Further, it may get wetted in the rain, and moisture may condense on it in the presence of large humidity, which may blur the user's vision. Therefore, the present study aims to improve the effectiveness of a face shield. Our measurements demonstrate that the face shield, coated by silica nanoparticles solution, becomes superhydrophobic and results in a nominal hysteresis to the underlying surface. We employ high-speed visualization to record the impact dynamics of microliter droplets with a varying impact velocity and angle of attack on coated and non-coated surfaces. While the droplet on non-coated surface sticks to it, in the coated surface the droplets bounce off and roll down the surface, for a wide range of Weber number. We develop an analytical model and present a regime map of the bouncing and non-bouncing events, parametrized with respect to the wettability, hysteresis of the surface, and the Weber number. The present measurements provide the fundamental insights of the bouncing droplet impact dynamics and show that the coated face shield is potentially more effective in suppressing the airborne and fomite transmission.

As of October 2021, the total reported global deaths due to COVID-19 is around 4.8 × 10^6^,^1^ which serves as a grim reminder for humankind as well as scientists to radically innovate scientific solutions that help to mitigate the spread of the disease. The coronavirus is transmitted via respiratory droplets exhaled by an infected individual, a fact that is widely accepted.^2–5^ The disease transmission is predominantly airborne, wherein the susceptible is directly exposed to the respiratory droplets exhaled by the infected individual.^6,7^ It may also be caused by virus-laden tiny aerosolized droplets ( ≤5 μm) that undergo slower evaporation and remain suspended for an extended time in the air, typically responsible for the spread of the infection in closed spaces with poor ventilation.^8–11^ In addition, these droplets may deposit on the surfaces to form fomites,^12,13^ and the surface may remain contaminated with the virus for days.^14,15^

Measures like hand hygiene and fabrication of antiviral surfaces could reduce the risk of catching infection via the fomite route.^16,17^ The use of face masks has been a widespread practice to mitigate the disease spread by airborne route.^18,19^ The efficiency of face-covering has been demonstrated based on the reduction of cough cloud volume that entrains the surrounding air when exhaled in a closed space^20,21^ and the atomization of coughed droplets while penetrating with multi-layered masks.^22,23^ Researchers have devoted attention to improving the effectiveness and durability of masks. For example, recharging and rejuvenation of decontaminated N95 masks were experimentally demonstrated.^24^ Apart from masks, vacuum helmets^25^ and face shields^26–28^ are also used as protective measures to inhibit exposure to respiratory droplets. The role of the face shield becomes important in settings where physical proximity becomes unavoidable, such as in airplane cabin, elevator, and hospital settings. Especially, this protective shield prevents direct attachment of coughed or sneezed droplet on the face of the susceptible. Hence, improving the effectiveness of the face shield becomes imperative.

Both experimental and numerical studies have been reported to study the dynamics of respiratory droplet cloud around these protective shields and their effective design.^25–28^ The interactions of a water droplet, as a surrogate to a respiratory droplet, with face mask and solid surface have been studied. For instance, the virus survival on different surfaces have been explored by studying drying of droplets and residual thin-films.^14,15,17^ Penetration/blocking, secondary atomization, and aerosolization of droplets have been studied while interacting with masks.^22,23^ Yu *et al.*^29^ recently demonstrated an optically transparent coating technique based on cosmetic ingredients that is capable of capturing contaminated droplets from the air. While such a technique may remove infected aerosols from air, the surface may become a source of the fomite itself, which may remain a viable source of infection spread for an extended time. Thus, the effectiveness of the protective shields would be enhanced by inducing pathogen-containing droplet repellency onto their surfaces. In addition, the optical transparency should be kept intact for clear visibility through the surface. Therefore, the objective of the present work is to evaluate a transparent surface-coating that repels the airborne droplets, and this proposed technique has been demonstrated as a better way to keep the shield safe from fomite transmission while preventing the airborne transmission using the shield.

Repellency of the surface of the face shield against respiratory droplets is characterized by reduced adhesion between solid and liquid molecules. The solid–liquid adhesive strength determines the wettability of the solid surface by the liquid, which is characterized by the contact angle and contact angle hysteresis. The latter provides resistance against the motion of the triple-phase contact line over the solid surface.^30,31^ These are the factors that are responsible for the ultimate fate of an impacting droplet on the solid surface: an impacting droplet may equilibrate on the surface or may bounce off the surface. There have been significant efforts to tune the droplet-bouncing phenomenon and understanding of the underlying physics in terms of impact velocity, wettability, and energy dissipation.^32–39^ In conjugation with wettability, the kinetic energy of the impacting droplet also plays a crucial role in determining the sticking/bouncing process.^32–39^ The kinetic energy with respect to the droplet's surface tension and viscosity is determined by the dimensionless Weber (*We*) and Reynolds (*Re*) numbers (defined later in this Letter in the context of Figs. 3 and 4). Studies have revealed that for hydrophilic surfaces, at low *We* the impacting droplet first spreads over the surface and thereafter equilibrates on the surface. At high *We*, splashing and formation of fingering patterns at the droplet periphery at the maximum spread are seen.^40^ On the other hand, depending upon the *We*, an impacting droplet may rebound partially or completely from a hydrophobic surface. Interestingly, Richard and Quéré^33^ observed that complete bouncing can be achieved on microtextured superhydrophobic surfaces for sufficiently low We∼1. At high *We*, droplet elongation, necking, and ejection of secondary droplets are observed, which have been attributed to the excess available rebound kinetic energy that remains after part of the rebound energy is transformed into the droplet's intrinsic surface energy.^39,41^

While microtexturing of a low-energy surface induces water repellency,^33,42^ this process is not well-suited to manufacture a low-cost face shield. Moreover, while previous studies imparted valuable understanding to account for the effect of intrinsic wettability to predict droplet bouncing/sticking, the effects of dissipative forces were taken only to the first order.^32,43^ The knowledge of main dissipating mechanisms and their role in deciding whether a droplet bounces off the surface is still lacking. Gaining insights on the dissipative forces is crucial because it was observed^33^ that even for textured superhydrophobic surfaces, at high impact velocities; while viscous dissipation can be neglected, even a few degrees of hysteresis results in a significant reduction in the coefficient of restitution of a bouncing droplet. Hence, the effect of hysteresis dissipation in determining droplet bouncing remains a fundamental research question. This is particularly relevant in the context of designing effective face shields, because hysteresis depends not only on the physical surface texture, but also on the surface energy: even nanometrically smooth surface may exhibit significant hysteresis,^44^ which may enhance droplet sticking, thereby inhibiting the effectiveness of a face shield. Hence, a consolidated knowledge on how the droplet sticking and bouncing phenomena can be deciphered under a universal regime in terms of wettability, contact angle hysteresis, impact velocity, and droplet diameter is an urgent need.

In this Letter, we present the combination of proof-of-concept measurements and analytical approaches to demonstrate the proposed technique. It is a widespread practice to use the pure water droplets as surrogate to the respiratory droplets for laboratory and numerical simulation of droplet interactions with solid surfaces, e.g., masks.^14,15,17,22,23^ Hence, in accordance with the available literature, herein we seek to investigate an impacting water droplet's interaction with a surface containing optically transparent, superhydrophobic, and low contact angle hysteresis coating at different impact energies and angles to demonstrate the enhanced effectiveness of the designed protective shields. The following research questions are posed: (i) can an optically transparent surface coating technique be adapted to make the underlying surface repellent to respiratory droplets? (ii) What are the governing rules that dictate the performance of the coated surface? (iii) What is the role of the main dissipating forces that decide the eventual fate of the droplet? Fundamental knowledge of the physical mechanism of droplet sticking or bouncing off the surface would help to expand the proposed method's applicability: one may flexibly choose the optimized parameters found in this study to obtain the desired performance. The coating proposed herein can be applied irrespective of the underlying surface's composition, and in addition to repel respiratory droplets, such coating would also impede wetting of the surface in the rain, fogging of the surface in cold and humid weather, thereby retaining the visibility of the protective shield to the maximum extent. The process is simple and cheap to use, and can even be applied repeatedly on the same surface.

Figure 1 schematically shows the concept of proposed technique in which the droplet bounces off the coated face shield. Commercially available face shields are made of polyethylene terephthalate (PET),^26–28^ which is hydrophilic in nature^45^ and also corroborated by our measurements presented later. Further, it will be shown that the surface exhibits large contact angle hysteresis, and therefore, respiratory droplets impacting on the surfaces linger on it [cf. Fig. 1(a)]. Hence, commercially available face shields are sticky to respiratory droplets, including water. The applicability is, therefore, limited by an enhanced risk of fomite transmission, wetting in the rain, and fogging in cold and humid weather. In contrast, the droplets bounce off or roll down on the coated surfaces [cf. Fig. 1(b)] designed herein, which overcomes the aforementioned limitations. Based upon the observations, we develop an energy balance-based analytical model that includes all the relevant parameters—the intrinsic wettability, contact angle hysteresis, and impact momentum with respect to surface tension and viscosity—to depict regimes of bouncing and non-bouncing across varied impact energies. This way, the flexibility and expanded applicability of the process are presented.

**FIG. 1.**
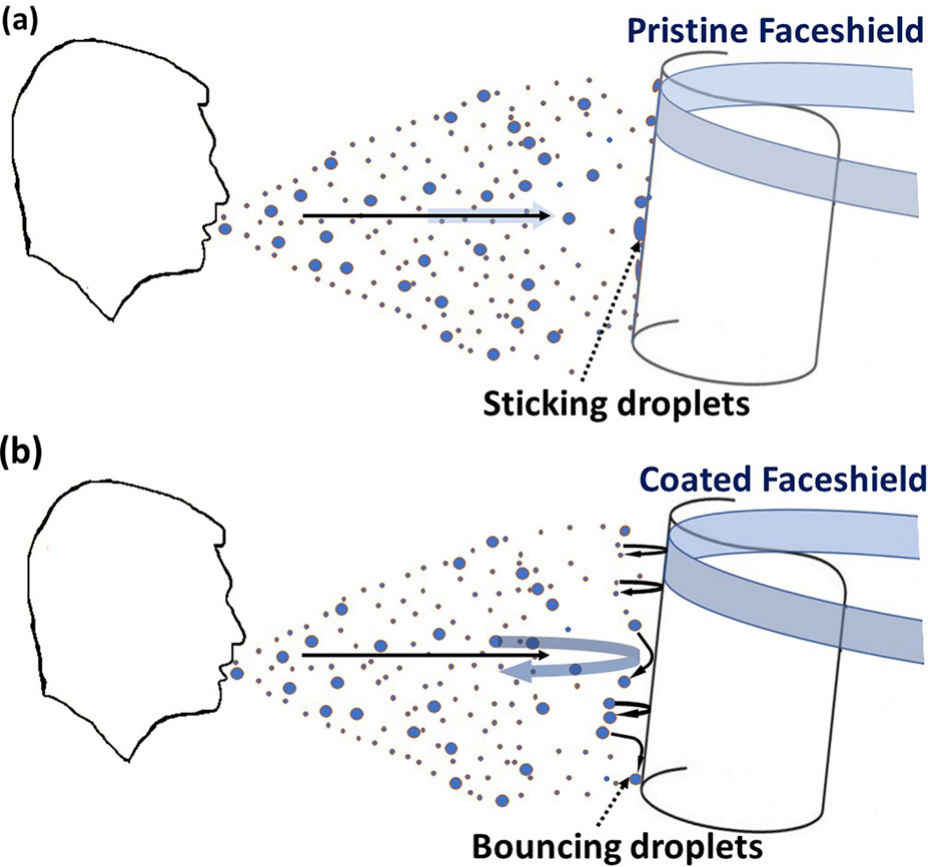
Evaluation of the intervention proposed in the present study to suppress the airborne and fomite transmission. (a) Respiratory droplets interact with a pristine face shield, wherein the droplets attach to the face shield surface after impact, and (b) respiratory droplets interaction with a face shield containing transparent, superhydrophobic, low contact angle hysteresis surface coating, wherein the droplets either bounce off or slide past the surface.

First, we present the methodology employed in the present study. The details of the experimental procedure are provided in Secs. S1–S3 of the supplementary material. At the heart of this study (cf. Fig. S1 of the supplementary material) is the coating of a face shield surface with Glaco Mirror Coat^TM^ (henceforth will be called as coated surface), which is an alcohol-based suspension of perfluorinated silica nanoparticles, that induces superhydrophobicity by depositing silica nanoparticles (∼40 nm) onto the surface, i.e., the particles are physisorbed.^46–48^

Figure 2 shows the surface characterization for pristine and coated surfaces. Figure 2(a-i) depicts a representative view of sessile de-ionized (DI) water deposited on pristine face shield surface. The apparent contact angle was measured to be $\theta_{\mathrm{app}}=74.5{}^{\circ}\pm 1{}^{\circ}$, which is consistent with published *θ_app_* measurements on PET^45^ and hence hydrophilic. The contact angle hysteresis measurements by tilting plate method [cf. Fig. 2(a-ii) for a representative image] revealed an advancing contact angle $\theta_{\mathrm{adv}}=80{}^{\circ}\backslash ,\pm \backslash ,2{}^{\circ}$ and receding contact angle $\theta_{\mathrm{rec}}=30{}^{\circ}\backslash ,\pm \backslash ,2{}^{\circ}$. The gravity-driven droplet roll-off angle was observed to be $\alpha_{s}∼75{}^{\circ}$. Such an order of hysteresis is desired even on surfaces having nanometric roughness, which is attributable to a residual thin-film left behind the receding triple-phase contact line.^44^ This notion is further reinforced by the present observation of $\theta_{\mathrm{app}}\approx \theta_{\mathrm{adv}}$.^44^ Figures 2(a-iii) and 2(a-iv) depict the representative atomic force microscopy (AFM) image and the corresponding perspective view of the pristine face shield. Measurements reveal a root-mean-square (RMS) roughness of ∼0.37\,±\,0.1 nm, and an area %∼ 0.072, which returns a surface area factor^44^ *r *=* *1.000 72. Hence, the surface is having a nominal roughness. The hydrophilicity ensues from the higher affinity of the surface to water, and hence the degree of liquid film retention behind the triple phase contact line is higher, which is responsible for the significant hysteresis of $\Delta \theta_{H}=50{}^{\circ}\backslash ,\pm \backslash ,2{}^{\circ}$ observed in Fig. 2(a-ii). Therefore, the sticky nature of face shields to droplets containing maximum portion of water, which is the case of respiratory fluids, is experimentally proven. Figure 2(a-v) depicts a representative view of optical transparency test of a pristine face shield surface. The optical transparency test was conducted by viewing through the face shield the acronym “IITB,” which shorts for our institute name “Indian Institute of Technology Bombay;” printed on a white paper for the present experimental purpose. Observations reveal a high degree of optical transparency, which is expected for a face shield.

**FIG. 2.**
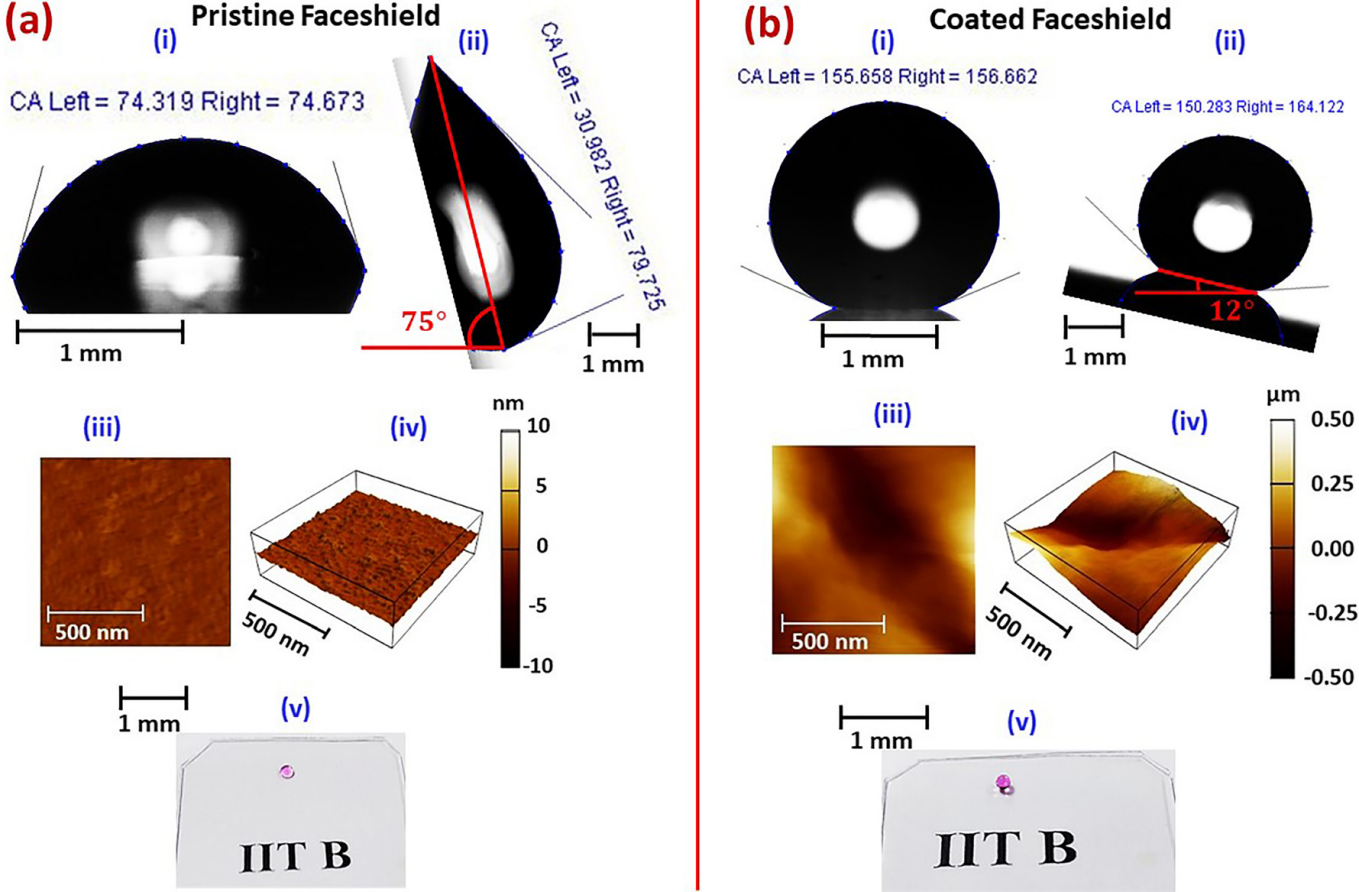
Surface characterization of pristine and coated face shield. (a) Pristine face shield: (i) apparent contact angle, (ii) contact angle hysteresis, (iii) AFM image, (iv) perspective view of the AFM image depicted in (iii) and (v) optical transparency test; (b) coated face shield: (i) apparent contact angle, (ii) contact angle hysteresis, (iii) AFM image, (iv) perspective view of the AFM image depicted in (iii), and (v) optical transparency test. In [a (v)] and [b (v)], “IITB “shorts for” Indian Institute of Technology Bombay”; our institute name. The acronym, printed on a white paper, was observed through the pristine and coated face shields in order to examine their optical transparency. Simultaneously, 1 *μ*l water droplets dyed with potassium permanganate (violet color) were deposited on the sample surfaces which show a reduced wettability of the coated face shield, consistent with [a (i) and (ii)] and [b (i) and (ii)].

Figure 2(b-i) shows a representative view of *θ_app_* measurements on coated face shield. Figure 2(b-ii) depicts a representative view of contact angle hysteresis measurements by tilting plate method. We measure that *θ_app_*, *θ_adv_*, *θ_rec_*, $\Delta \theta_{H}$, and *α_s_* are 156.2°\,±\,2°, 164°\,±\,2°,150.2°\,±\,4°, 14°\,±\,2°, and ∼12°, respectively. Hence, coating on face-shield surface induces superhydrophobicity and significantly reduces the hysteresis, i.e., the surface becomes lesser adhesive to the water molecules, and the droplets deposited on it would roll down under gravity for a slight inclination of the surface. Hence, the surface could be thought to be water repellent. Later on, we will further demonstrate this repellency by conducting droplet bouncing experiments at different impact energies and angles. The surface roughness measurements by AFM [cf. Figs. 2(b-iii) and 2(b-iv) for representative views] revealed an RMS roughness of 127 ± 10 nm and area %∼ 134.4 with corresponding *r *=* *2.334. The significant corrugation observed in the coated surface can be attributed to the deposition of silica nanoparticles as discussed earlier. The nanoparticles possibly create air pockets beneath the sessile droplet which, along with their chemical nature induces the observed superhydrophobicity,^46–48^ and the discontinuity in the contact line is responsible for the reduced hysteresis.^49^ Figure 2(b-v) shows the optical transparency test of the coated face shield in the same fashion as was conducted for pristine face shield shown in Fig. 2(a-v). A comparison between Figs. 2(a-v) and 2(b-v) clearly shows that no deterioration in optical transparency occurs due to coating on the face shield surface. In order to delineate the enhanced superhydrophobicity on the coated face shields while retaining the optical transparency, we deposited a 1 *μ*l de-ionized water droplets dyed with potassium permanganate (violet color) on the surfaces while capturing the images of Figs. 2(a-v) and 2(b-v). Clearly, the preserved optical transparency and the enhanced non-wetting character of the coated surface are realized. The enhanced non-wetting character for the coated surface seen in Figs. 2(a-v) and 2(b-v) is consistent with the observations of Figs. 2(a-i) and 2(a-ii) and 2(b-i) and 2(b-ii). A summary of surface characterization parameters of the pristine and coated face shield surfaces is depicted in Table I.

**TABLE I.** Summary of surface characterization parameters (cf. Fig. 2).

Surface	$\theta_{\mathrm{app}}{(}^{{}^{\circ}})$	$\Delta \theta_{H}{(}^{{}^{\circ}})$ and $\alpha_{s}{(}^{{}^{\circ}})$	RMS roughness (nm)	Area percent
Pristine	74.5 ± 1	50 ± 2 and 75	0.37 ± 0.1	0.072
Coated	156.2 ± 2	14 ± 2 and 12	127 ± 10	134.4

In order to further decipher the water repellency, and thereby the enhanced effectiveness of the coated face shield, we investigate the impact of the droplet on the pristine and coated surfaces under different impact velocities and angles. The experimental parameters are detailed in the supplementary material. The droplet diameter and range of impact velocity in the experiments were 1.7 ± 0.05 mm and [0.2–1.39] m/s (obtained by varying the impact height ∈[0.3,10] cm), respectively. The non-dimensional numbers—Weber number (*We*) and Reynolds number (*Re*)—are defined as follows: $We=\rho_{L}V_{0}^{2}D_{0}/\gamma_{L}$ and $Re=\rho_{L}V_{0}D_{0}/\mu$, where $\rho_{L}=1000$ kg/m^3^ is the density of water, *V*_0_ is the impact velocity, *D*_0_ is the initial droplet diameter before the impact, μ=0.00 089 Pa-s is the dynamic viscosity of water, and *γ_L_* = 0.072 J/m^2^ is the surface tension of water. We vary *We* in the range of [0.94, 45.6] to delineate the impact dynamics, and the correlation between *We* and *Re* is plotted in Fig. 3.

**FIG. 3.**
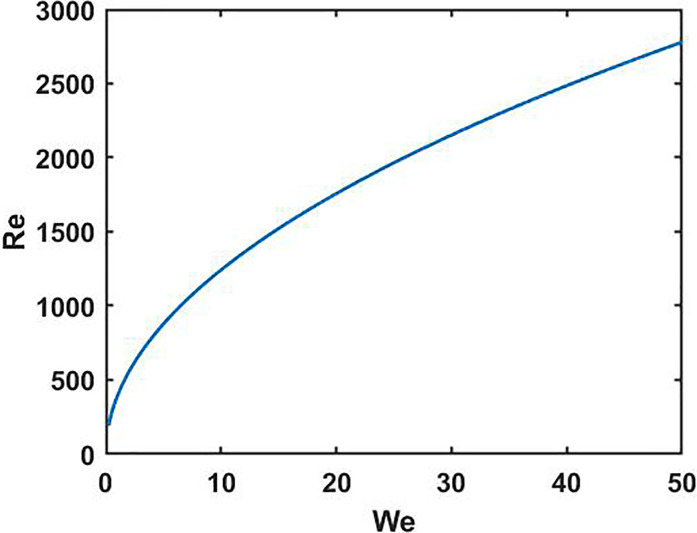
Correlation between *We* and *Re* for a wide range of We∈[0.1,50]. The experiments are conducted in the Weber number range of [0.94, 45.6].

Further, we provide a mapping of the present experiments of surrogate water droplets to realistic scenarios of impact of respiratory droplets on a face shield and plot iso-contours of Weber number (*We*) in droplet diameter (*D*_0_)—impact velocity (*V*_0_) plane in Fig. 4(a). Since the droplet bouncing or non-bouncing depends on *We* and contact angle of the surface, the present experiments along with Fig. 4(a) can be used to predict respiratory droplet fate (bouncing or sticking) on the coated face shield. Diameters and impact velocity in realistic scenarios of impact of respiratory droplets are given as follows. Typically, 95% of coughed, sneezed, or speech droplets are in range of 2–200 *μ*m.^50–54^ The typical ranges of velocity in indoor spaces,^55^ outdoors,^56^ cough wave front velocity,^57^ and aircraft cabin^58^ are [0.05–0.8], [0.5–3.1], [0.1–6], and [0.1–0.5] m/s, respectively [Fig. 4(b)]. Therefore, the impact of aerosols (*D*_0_ < 5 *μ*m) or respiratory droplets (5 < *D*_0_ < 200 *μ*m) in several possible scenarios can be predicted using the present experiments. Further, the range of *We* chosen to study the coated face shield's repellency against respiratory droplets will also include its repellency against rain drops. For natural rainfall, the *We* varies in the range^59^ 50–1200, which is much higher than that considered in the present study. At higher *We*, the droplets bounce off a superhydrophobic surface more prominantly,^33,40,41^ a convention which also corroborates with our experiments and model [cf. Fig. 10(b)]. Hence, the coated surfaces developed to repel respiratory droplets will automatically repel rain drops as well. Further, the low hysteresis and nominal droplet roll-off angle [ ∼12°, cf. Fig. 2(b)] observed on coated face shield surface are advantageous in inhibiting fogging in cold and humid weather: droplets stemmed from it will roll down from the surface worn by the user in a vertical orientation. This way, the parameters chosen in this study cover a wide spectrum of applicability of the coated face shields, which will be evident further from the model comparisons with experiments [cf. Fig. 10(b)].

**FIG. 4.**
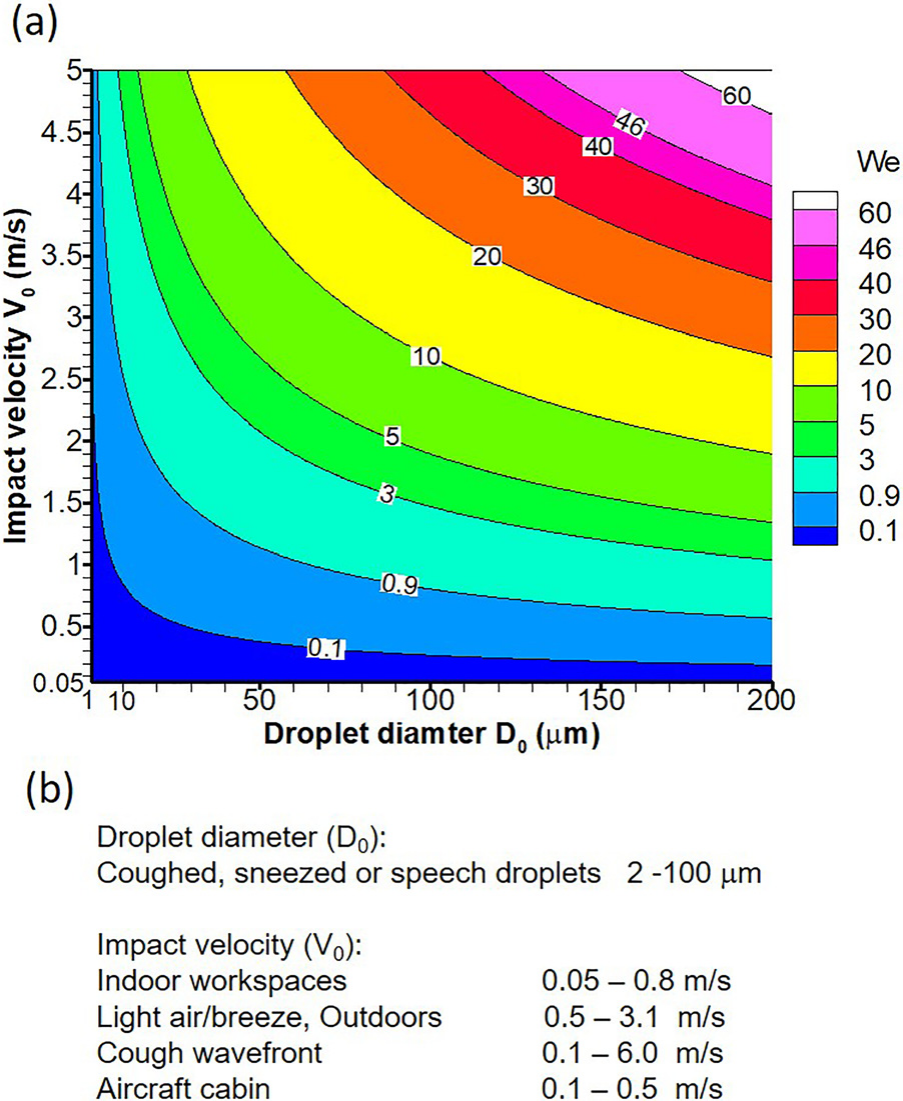
(a) Iso-contours of Weber number (*We*) in droplet diameter (*D*_0_)—impact velocity (*V*_0_) plane. Minimum and maximum values of *We* in the present experiments are shown as iso-contours of *We *=* *0.9 and *We *=* *46. (b) Droplet diameters and impact velocity in realistic scenarios of impact of respiratory droplets on a face shield. Sources of these data are given in the text.

Second, the orthogonal impact of the droplet on the pristine and coated face shield surfaces is presented. Figure 5 shows the time frames of droplets impacting on the pristine [Fig. 5(a)] for *We *=* *0.94, Fig. 5(b) for *We *=* *45.6] and coated [Fig. 5(c) for *We *=* *0.94, Fig. 5(d) for *We *=* *45.6] face shield surfaces. The typical features of impact dynamics are as follows. After the impact, the droplet spreads over the surface to attain a maximum spreading wetted diameter *D_max_*. Thereafter, the droplet recoils. For pristine face shield [cf. Figs. 5(a) and 5(b)], after recoil, the droplet does not bounce off even for *We *=* *45.6, the highest *We* considered. The free surface of the droplet first oscillates due to the competition between the surface and kinetic energy, and thereafter, the droplet equilibrates on the surface. In contrast, for the coated superhydrophobic surface [cf. Figs. 5(c) and 5(d)], the droplet bounces off the surface after recoil even for the lowest *We *=* *0.94. The details of the physical mechanism of bouncing and the energies involved at different stages will be discussed later using an analytical model.

**FIG. 5.**
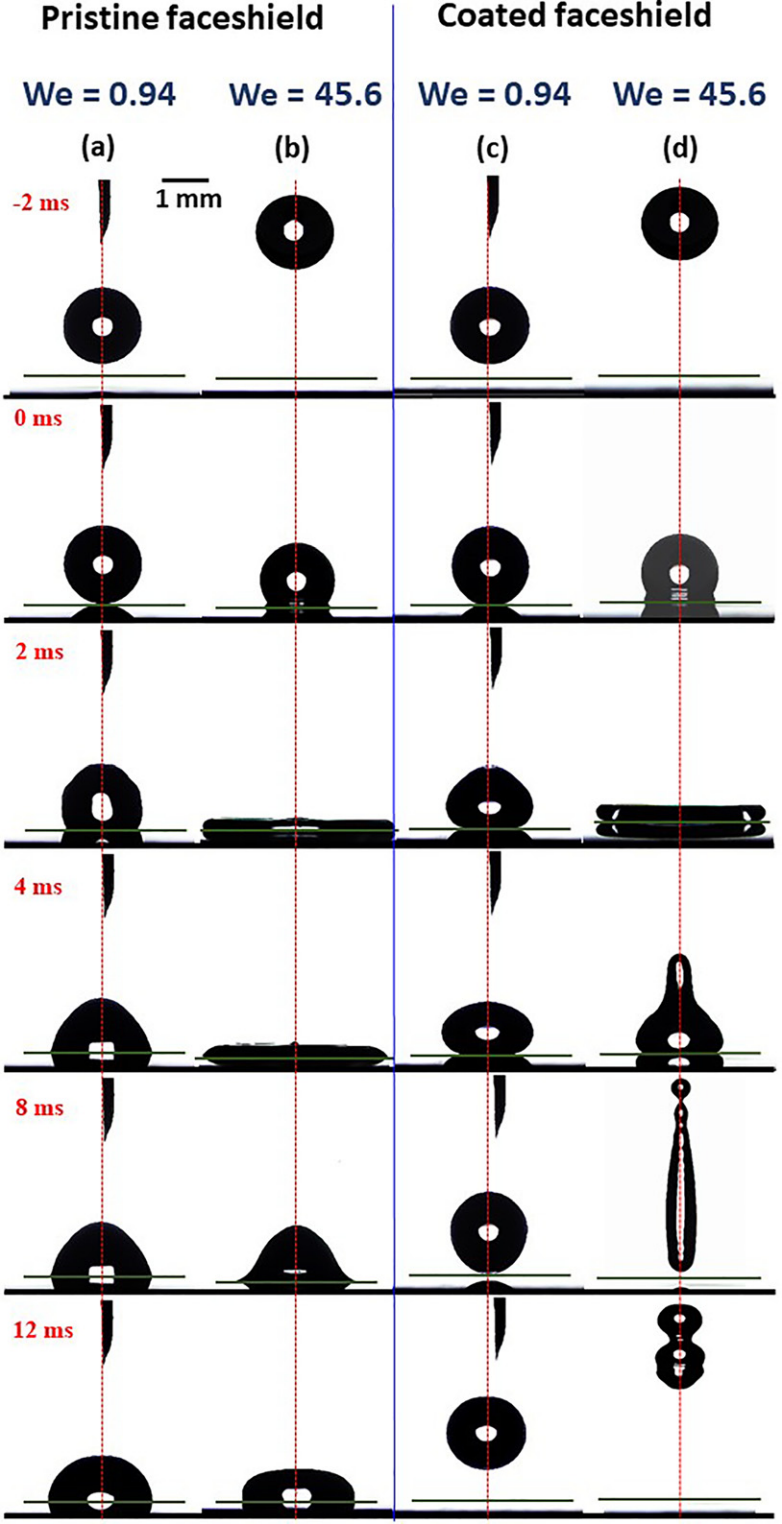
Time frames of droplets impacting on the surfaces: (a) on pristine face shield, at *We *=* *0.94, (b) on pristine face shield, at *We *=* *45.6, (c) on coated face shield, at *We *=* *0.94, and (d) on coated face shield, at *We *=* *45.6. Multimedia view: https://doi.org/10.1063/5.0073724.1
10.1063/5.0073724.1

Figure 6 shows a plot depicting the time variation of wetted diameter *D*(*t*), normalized by the initial diameter *D*_0_ for different *We* considered in the present study. For pristine face shield [cf. Fig. 6(a)], maximum spreading is attained at time ∼1.8–4.5 ms. After recoil, the droplet equilibrates on the surface with a final $D(t)/D_{0}\approx 1.4$. For the coated face shield [Fig. 6(b)], maximum spreading is attained between 1.25 and 2.75 ms. After recoil, the droplet *completely* bounces off. The total contact time of the droplet with the surface is estimated to be around 7.5 ms.

**FIG. 6.**
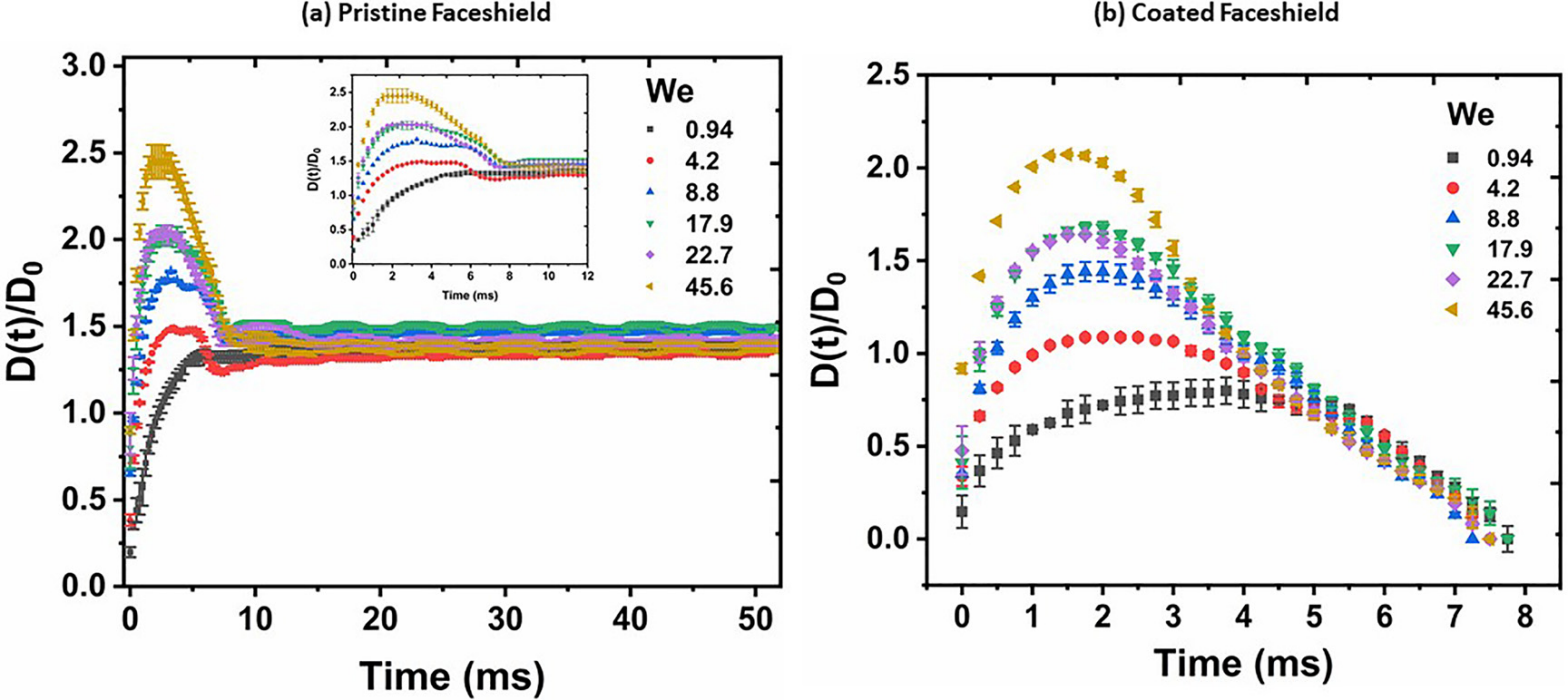
Instantaneous wetted diameter, *D*(*t*), normalized with respect to the initial droplet diameter, *D*_0_, for different *We*. (a) Pristine face shield—inset shows a magnified view around *D_max_* and (b) coated face shield. Representative error bars are also shown in the figure.

Third, we present the effect of varying impact angle on the impact dynamics of water droplets on the pristine and coated face shields at different *We*. To vary the impact angle, the angle of inclination, *α*, of the surface with respect to the horizontal plane was varied in range of [0^°^,70^°^]. Figure 7(a) shows the variation of $D_{\text{max}}/D_{0}$ with respect to *α* for different *We* for the pristine face shield surface. Droplet side views at different time frames for a representative α=40° are also shown for *We *=* *0.94 and *We *=* *45.6. For a given *α*, $D_{\text{max}}/D_{0}$ increases with *We*, which is expected as higher *We* provides higher available kinetic energy for spreading.^60^ This is consistent with the observation in Fig. 6, wherein *α* was set to zero. However, $D_{\text{max}}/D_{0}$ exhibits an increasing trend with increase in *α*. This is a consequence of higher hysteresis reported in Fig. 2(a). At higher *α*, the component of velocity along the surface is higher, which forces the droplet to slide down the surface; however, the sliding is opposed by the hysteresis resistance, which augments the solid–liquid contact area and an increased $D_{\text{max}}/D_{0}$ is realized. Further, we quantify the impact dynamics on the coated face shield under varying *α*. Nonetheless, the droplet never bounces off the surface, even at the highest α=70° and *We *=* *45.6, and equilibrates on the surface with different wetting angles at the front and rear edges, consistent with the contact angle hysteresis [cf. Fig. 2(a)]. This further shows the sticky nature of commercially available face shield surfaces to droplets.

**FIG. 7.**
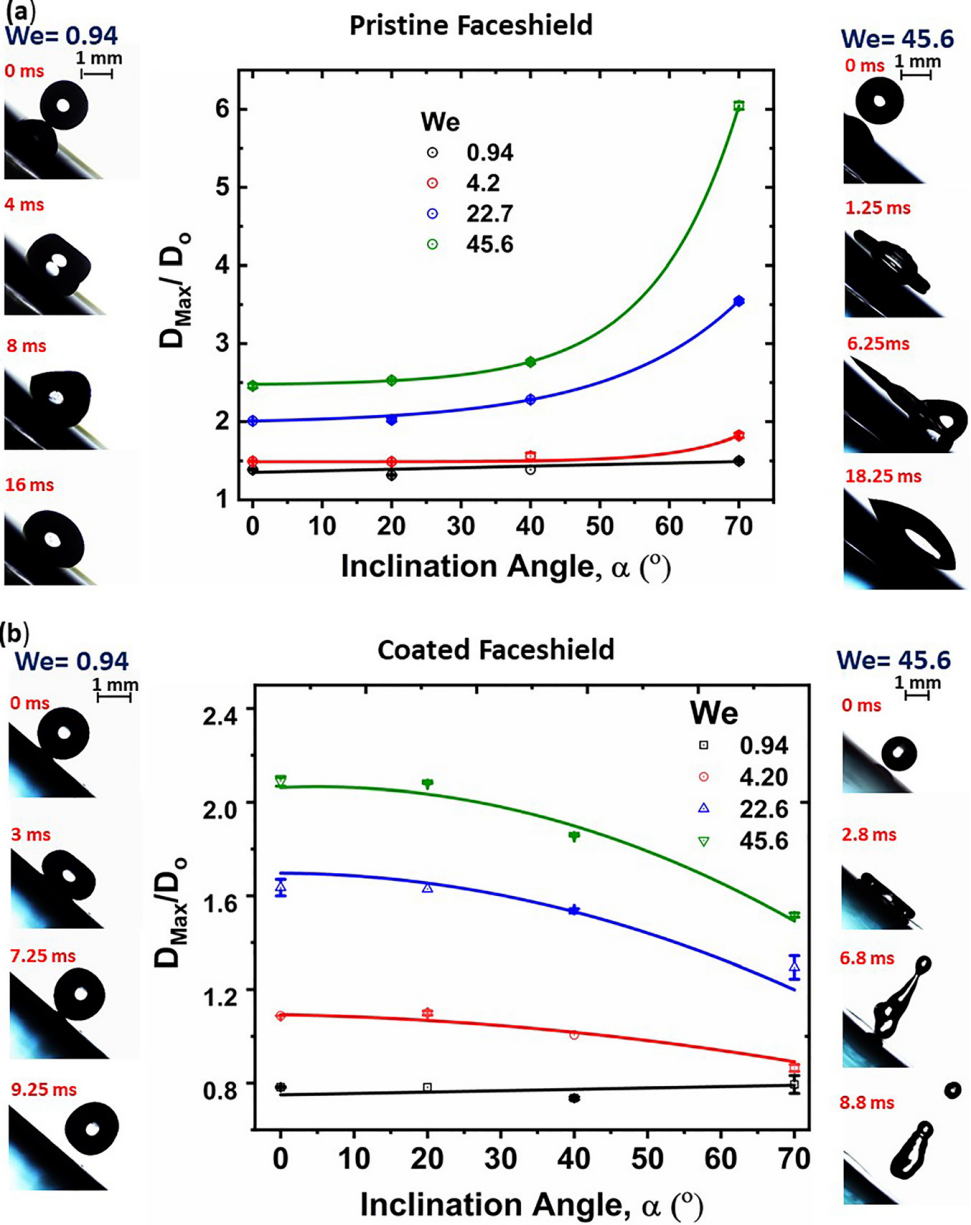
Variation of $D_{\text{max}}/D_{0}$ with the surface inclination angle, *α*, of the surface at different *We.* (a) Pristine face shield and (b) coated face shield. Representative droplet images at different time instances for α=40° are shown for *We *=* *0.94 (left column) and *We *=* *45.6 (right column). Multimedia view: https://doi.org/10.1063/5.0073724.2
10.1063/5.0073724.2

Figure 7(b) shows the results for the coated face shield. $D_{\text{max}}/D_{0}$ increases with *We*, which is expected, as was also seen in Fig. 7(a) for the pristine face shield. Interestingly, for the case of coated samples, $D_{\text{max}}/D_{0}$ decreases with *α*, which is in stark contrast to the case of pristine samples [cf. Fig. 7(a)]. This is a consequence of the reduced hysteresis of the coated face shield surfaces [cf. Fig. 2(b)]. At higher *α*, the component of impact velocity parallel to the surface increases; and due to less hysteresis, the triple-phase contact line experiences less resistance in its motion and the droplet rolls off, thereby decreasing $D_{\text{max}}/D_{0}$. For coated surfaces, the droplet rolls off and eventually bounces off the surface even for the lowest *We *=* *0.94. This marks the enhanced water repellency and effectiveness of the coated face shield.

From Figs. 6 and 7, we note the following general observations. At low *We *=* *0.94 for the case of pristine sample, the droplet does not have enough kinetic energy to overcome the solid–liquid adhesive energy, and therefore, after getting engaged with the surface, it gradually spreads to reach the “equilibrium” wetted diameter; hence, the spreading is *capillary driven.*^61^ At higher *We*, sufficient kinetic energy is available to overcome the adhesion, and therefore, the wetted diameter passes through a maxima after which the droplet recoils to the equilibrium diameter dictated by the solid–liquid adhesive strength. Before coming to static equilibrium, the liquid–vapor interface oscillates due to the competition between the kinetic and surface energy. Hence, at higher *We*, the spreading is *impact driven*. Conversely, for the case of coated samples, for all *We*, the wetted diameter passes through a maxima, and the droplet bounces off. This is because the droplet would always have enough kinetic energy to overcome the solid–liquid adhesive energy for a superhydrophobic surface, and after recoil, it has enough kinetic energy to regain its spherical shape.

The contact time of the droplet with a superhydrophobic surface scales as the capillary time given by^62,63^ $\tau_{\mathrm{cap}}=\sqrt{\frac{\rho_{L}D_{0}^{3}}{\gamma_{L}}}$. Figure 8 illustrates a plot between the droplet contact time and *We* at different *α* for coated samples. While the overall data lie around *τ_cap_*, consistent with previous reports on superhydrophobic surfaces,^62,63^ we observe a slightly higher contact time for the case of α=70°, and low *We* (=0.94). This is attributed to the fact that for higher inclination angle, the velocity component along the surface increases, while that orthogonal to the surface decreases. Therefore, available energy for rebound is lesser; however, the droplet tends to slide down, which stems from the reduced hysteresis on coated surfaces. At higher We (≥4.2), the data for α=70° almost coincide with those of α=0°,20°, and 40°. We mention here that for the case of α=70° and *We *=* *45.6, the droplet went out of the experimental window during the recoil, slide, and rebound. Hence, the total contact time data could not be recorded and is not presented in Fig. 8. We observe a slightly decreasing trend of the contact time with increasing *We*. However, apart from the case of *We *=* *0.94 and α=70°, the decay in the contact time remains within 20% and hence is not significant. Overall, the contact time remains in the same order of magnitude as *τ_cap_* for all *α* and *We*. The almost similar contact time for varied *α* is favorable to suppress chances of COVID-19 infection since coughed or sneezed droplets could impact on the face shield at different angles.

**FIG. 8.**
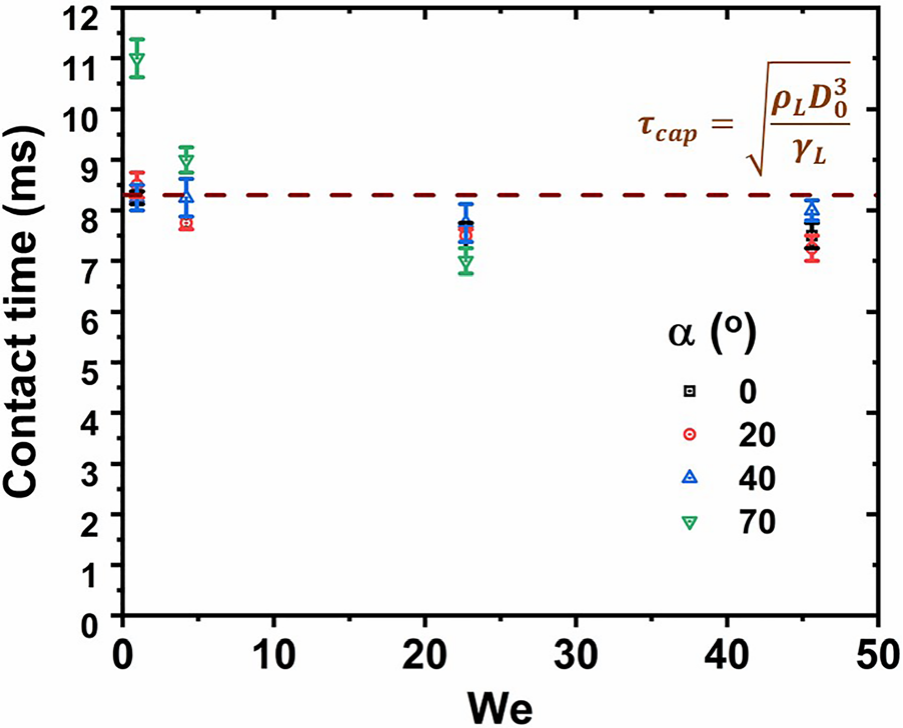
Contact time of the droplet impacting on coated face shield as a function of *We*, at different *α*.

Fourth, we present a model to predict the experimentally observed bouncing and non-bouncing phenomena on the coated and pristine surfaces, respectively. In conjugation with the experiments, we present a regime map of bouncing and non-bouncing phenomena in terms of crucial parameters, such as *θ_app_*, $\Delta \theta_{H}$, and $D_{\text{max}}/D_{0}$, which would help expanding the applicability of the process: one may choose the optimized parameters of the surface coating to achieve the desired performance. Figure 9 schematically shows the spreading and recoil during impact, which is consistent with the experimental observation (cf. Figs. 5–7). A droplet of diameter *D*_0_ first engages with the surface (stage a) with an initial kinetic energy, which is proportional to *We*, as for a homogeneous, one component liquid, *γ_L_* is a constant. After impact, the kinetic energy is converted to surface energy, owing to which the droplet spreads over the surface and attains a maximum wetted diameter, *D_max_* (stage b).^32,60^ Thereafter, the droplet recoils (stage c) and the surface energy is converted back to kinetic energy, which provides the *excess rebound energy*. According to previous studies,^32,60^ if the excess rebound energy exceeds the droplet's initial surface energy and gravitational energy, the droplet will have sufficient energy to regain its spherical shape and will bounce off, otherwise not. Mao *et al.*^32^ gave a mathematical expression for the bouncing criteria. According to that, the droplet will bounce if the excess rebound energy normalized with respect to the droplet's initial surface and gravitational energy, $E_{\mathrm{ERE}}^{*}>0$. In Figs. 5–7, it was observed that the impacting droplet always bounce off the coated face shield surfaces. After recoil, when the droplet bounces off the surface, the droplet deforms and part of the excess rebound energy is spent to regain its surface energy and thereby the spherical shape. At higher *We* (= 45.6), there remains enough excess rebound kinetic energy to overcome the droplet's intrinsic surface energy, which leads to an elongation of the bouncing droplet. Consequently, a necking region originates at the top of the elongated droplet from where a secondary droplet is ejected as visible from the image frames (cf. Figs. 5–7). Such a breakup of bouncing droplets on superhydrophobic surfaces was observed earlier as well.^39,41^ However, eventually, the underlying coated face shield surface remains dry after the droplets impact and rebound, hence fulfilling the principle goal of this research.

**FIG. 9.**
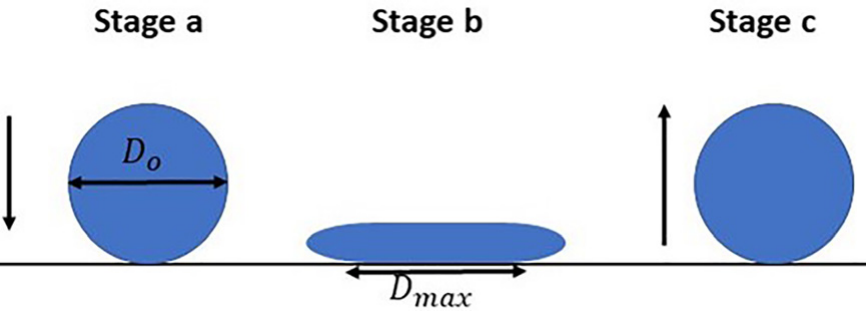
Schematic of the model considered herein showing the spreading and recoil of an impacting droplet on a surface.

In the intermediate stages of spread and recoil, the droplet experiences dissipation-induced energy loss. Mao *et al.*^32^ used an empirical expression for the dissipative energy based upon least-square data regression of their measurements. Herein, we attempt to find an exact expression for the dissipative energy. In accordance with the literature,^33^ we ignore the viscous dissipation because of the high *Re *>* *100 of the problem as depicted in Fig. 3. This is also true for droplets generated during respiratory events: previous studies considered similar and even higher *Re* for the purpose of laboratory and computational simulation of actual respiratory events.^22,64–67^ Hence, neglecting viscous dissipation is justified in the context of both the present experiments and respiratory events: the results for bouncing/non-bouncing regimes will automatically stay valid. The second component the dissipation stems from hysteresis, which cannot be ignored for a few tens of hysteresis values.^33^ Hence, we construct the dissipative energy term, *E_diss_* from hysteresis. The energy dissipated per unit area during an hysteresis cycle is given by^30^ $\Delta H=\gamma_{L}(\text{cos}\;\theta_{\mathrm{rec}}-\text{cos}\;\theta_{\mathrm{adv}})$. Hence, for spreading and recoil, the total energy dissipated reads as

$$E_{\mathrm{diss}}=\pi \frac{D_{\text{max}}^{2}}{4}\gamma_{L}(\text{cos}\;\theta_{\mathrm{rec}}-\text{cos}\;\theta_{\mathrm{adv}}).$$ (1)

In order to verify our hypothesis, we experimentally obtained the energy dissipated during spreading and recoil for the coated surfaces. We monitored the impact and rebound velocities for multiple impacts and bounces in a single experimental run (cf. Sec. S4 of the supplementary material for detailed methodology and calculations), and the energy dissipated per unit area, $\Delta \varepsilon_{\mathrm{diss}}$, was obtained from the coefficient of restitution,^33^ which was compared with ΔH. The comparison is shown in Fig. 10(a). As seen from Fig. 10(a), $\Delta \varepsilon_{\mathrm{diss}}$ data can be well approximated with ΔH obtained for coated face shield. Hence, the dissipation is mainly governed by hysteresis, i.e., Eq. (1). Plugging Eq. (1) into the expression for $E_{\mathrm{ERE}}^{*}$ given in Ref. 32 [cf. Eq. (26) therein], we obtain

$$\begin{array}{c} E_{\mathrm{ERE}}^{*}=\frac{1}{4}{\left(\frac{D_{\text{max}}}{D_{0}}\right)}^{2}(1-\text{cos}\;\theta_{\mathrm{app}})+\frac{2}{3}\frac{D_{0}}{D_{\text{max}}}\\ -\frac{1}{4}{\left(\frac{D_{\text{max}}}{D_{0}}\right)}^{2}(\text{cos}\;\theta_{\mathrm{rec}}-\text{cos}\;\theta_{\mathrm{adv}})-1 \end{array}.$$ (2)

wherein the bouncing criterion is given by: $E_{\mathrm{ERE}}^{*}>0$.

**FIG. 10.**
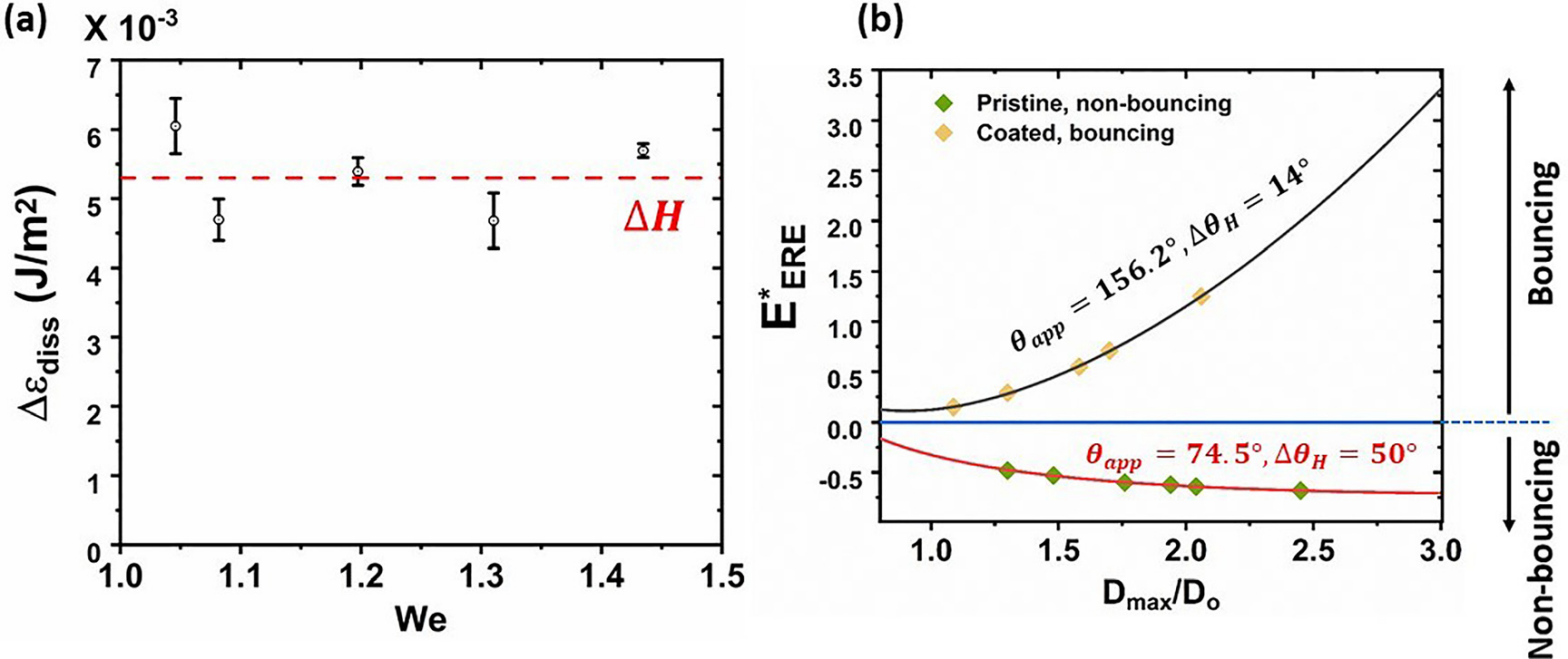
(a) Comparison between $\Delta \varepsilon_{\mathrm{diss}}$ and ΔH with varying *We*, and (b) regime map of bouncing/non-bouncing regimes parametrized with respect to *θ_app_*, $\Delta \theta_{H}$, and $\frac{D_{\text{max}}}{D_{0}}∼{\left(We\right)}^{\frac{1}{4}}$. Each symbol represents an experiment in terms of $D_{\text{max}}/D_{0}$, *θ_app_*, and $\Delta \theta_{H}$, and their color identifies the bouncing/non-bouncing regime they fall.

Figure 10(b) shows a plot between $E_{\mathrm{ERE}}^{*}$ and $D_{\text{max}}/D_{0}$. Herein, $D_{\text{max}}/D_{0}$ has been chosen as independent variable and has been varied over a large range to evaluate $E_{\mathrm{ERE}}^{*}=f(\frac{D_{\text{max}}}{D_{0}})$, as $D_{\text{max}}/D_{0}$ is a manifestation of impact conditions, such as *θ_app_*, *We*, and *Re*.^43^ Hence, a wide range of impact events will be well accommodated within the model framework. The diagram in Fig. 10(b) presents bouncing and non-bouncing regimes in the parameter space of $D_{\text{max}}/D_{0}$, *θ_app_*, and $\Delta \theta_{H}$. Each symbol represents an experiment in terms of $D_{\text{max}}/D_{0}$, *θ_app_*, and $\Delta \theta_{H}$, and their color identifies the bouncing/non-bouncing regime they fall. It is seen that pristine face shield surfaces always fall within non-bouncing regime, whereas the coated surface always falls in the bouncing regime. Therefore, a universal regime map for bouncing/non-bouncing phenomena has been proposed, which is parametrized with *θ_app_*, $\Delta \theta_{H}$, and $\frac{D_{\text{max}}}{D_{0}}∼{\left(We\right)}^{\frac{1}{4}}$, that expands the applicability of the proposed technique. We emphasize that, in actual respiratory events, the *We* or *Re* of the impacting droplets remains in the same range as considered herein, or even higher.^22,64–67^ Hence, neglecting the viscous dissipation and the regimes of bouncing for the case of the coated surface remain valid for real respiratory events.

In closure, we have demonstrated an improvement in the effectiveness of the protective face shield in the context of mitigation of spread of COVID-19. Commercially available face shields are made of polyethylene terephthalate (PET), which is hydrophilic and has high contact angle hysteresis, corroborated by the present measurements. Therefore, droplets impacting on the surface stick on it. They do not roll down on the surface and rather equilibrate on it. This enhances the risk of fomite transmission of the disease and also leads to wetting in rain and fogging in humid, cold weather. Herein, we demonstrate that face shield surfaces can be modified by silica nanoparticle-based optically transparent coatings, which induces superhydrophobicity and a nominal hysteresis to the underlying surface. Thereby, the impacting droplets will bounce off and roll down the surface. We have also developed an analytical model and presented a universal regime map of the bouncing and non-bouncing events, parametrized with respect to wettability, hysteresis, Weber number, and the Reynolds number to enhance the flexibility and expand the applicability of the present results and the proposed technique. The proposed solution of coating the face shield does not require any special expertise to coat the surface. The coating is commercially available at low cost and can be easily procured for day-to-day use. A face shield, therefore, with improved effectiveness may help in preventing the infection as well as reducing the spread of COVID-19.

## SUPPLEMENTARY MATERIAL

See the supplementary material for schematic and details of the experimental procedure, supporting results, and greater details of the analytical model.

## Data Availability

The data that support the findings of this study are available from the corresponding author(s) upon reasonable request.
